# Laparoscopic removal of an aberrant acupuncture needle in the gluteus that reached the pelvic cavity: a case report

**DOI:** 10.1186/s40792-020-01065-8

**Published:** 2021-02-17

**Authors:** Akira Yamamoto, Junichiro Hiro, Yusuke Omura, Takashi Ichikawa, Shozo Ide, Hiroki Imaoka, Hiromi Yasuda, Hiroyuki Fujikawa, Yoshiki Okita, Takeshi Yokoe, Masaki Ohi, Yuji Toiyama

**Affiliations:** Division of Reparative Medicine, Department of Gastrointestinal and Pediatric Surgery, Institute of Life Sciences, Mie University Graduate School of Medicine, 2-174 Edobashi, Tsu, Mie 514-8507 Japan

**Keywords:** Aberrant needle, Acupuncture, Laparoscopic surgery

## Abstract

**Background:**

Intrapelvic aberrant needles are rare in clinical practice. Long-term foreign bodies in the abdominal cavity may form granulation tissue or an abscess, and may cause organ injury. Therefore, such foreign bodies need prompt removal.

**Case presentation:**

A 26-year-old male athlete was referred to our hospital for investigation of an aberrant acupuncture needle in the gluteus. The needle was unable to be removed during acupuncture treatment, and the end broke off and remained in the gluteus. Abdominal X-ray examination showed a thin, 40-mm-long, metallic foreign body resembling an acupuncture needle. Abdominal computed tomography showed an abnormal shadow in the gluteus. However, it was unclear whether the tip of the needle reached the pelvic cavity. Thus, it was decided to surgically extract the needle via laparoscopic surgery under X-ray guidance as a safe and minimally invasive method. Although X-ray fluoroscopy confirmed that the aberrant needle was located in the gluteus, the needle could not be felt with the forceps, as the peritoneum surrounding the needle had granulomatous changes due to inflammation. Therefore, the retroperitoneum was further dissected to search for the needle. Once the needle was identified, its flexibility enabled it to be easily removed by grasping it directly with a needle holder. The length of the aberrant needle was 40 mm. The postoperative course was uneventful, and the patient was discharged from hospital on postoperative day 2.

**Conclusions:**

When a foreign body remains in the gluteus and its tip touches intrapelvic organs, such as the rectum, it is critical to determine the best approach for its safe removal. Given the anatomical location of the foreign body and the patient background, laparoscopic removal was considered the best approach in the present case.

## Background

Intrapelvic aberrant needles are rare in clinical practice, especially those caused by acupuncture treatment. Acupuncture is increasingly being integrated into conventional care for pain-related conditions [1]. However, some adverse effects such as organ injury, systemic infection, needle breakage, and forgotten needles have been reported [2]. An intrapelvic foreign body can occur due to remnants of medical procedures such as laparotomy [3], migration of orthopedic fixation devices [4, 5], perforation of the digestive tract after accidental ingestion [6], and introduction via the transvaginal [7], transurethral [8], transanal [9], and percutaneous routes. Long-term foreign bodies in the abdominal cavity may form granulation tissue or an abscess, and may cause organ injury [3]; therefore, prompt removal is required. We herein report a case of laparoscopic removal of an aberrant needle that was left in the gluteus muscle and migrated to the pelvis due to an accident during acupuncture treatment.

## Case presentation

A 26-year-old male athlete was referred to our hospital for investigation of an aberrant acupuncture needle in the gluteus. He had received acupuncture treatment 6 days before admission. During the acupuncture treatment, one treatment needle could not be removed, and the end broke off and remained in the gluteus. The practitioner tried to remove the needle immediately, but could not. Despite the foreign body in the gluteus, the patient did not stop training because there were no symptoms. He hated interruptions in training. He then presented with pain induced by flexion of the left lower limb, and was admitted to our department through orthopedics.

**Fig. 1:**
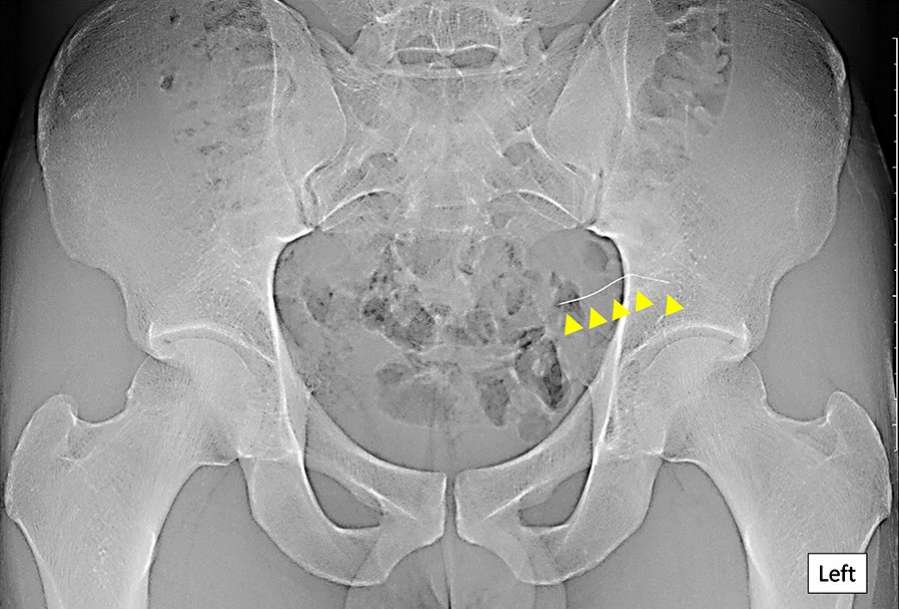
Abdominal X-ray examination on admission. A thin, 40-mm-long, metallic foreign body resembling an acupuncture needle is seen

**Fig. 2:**
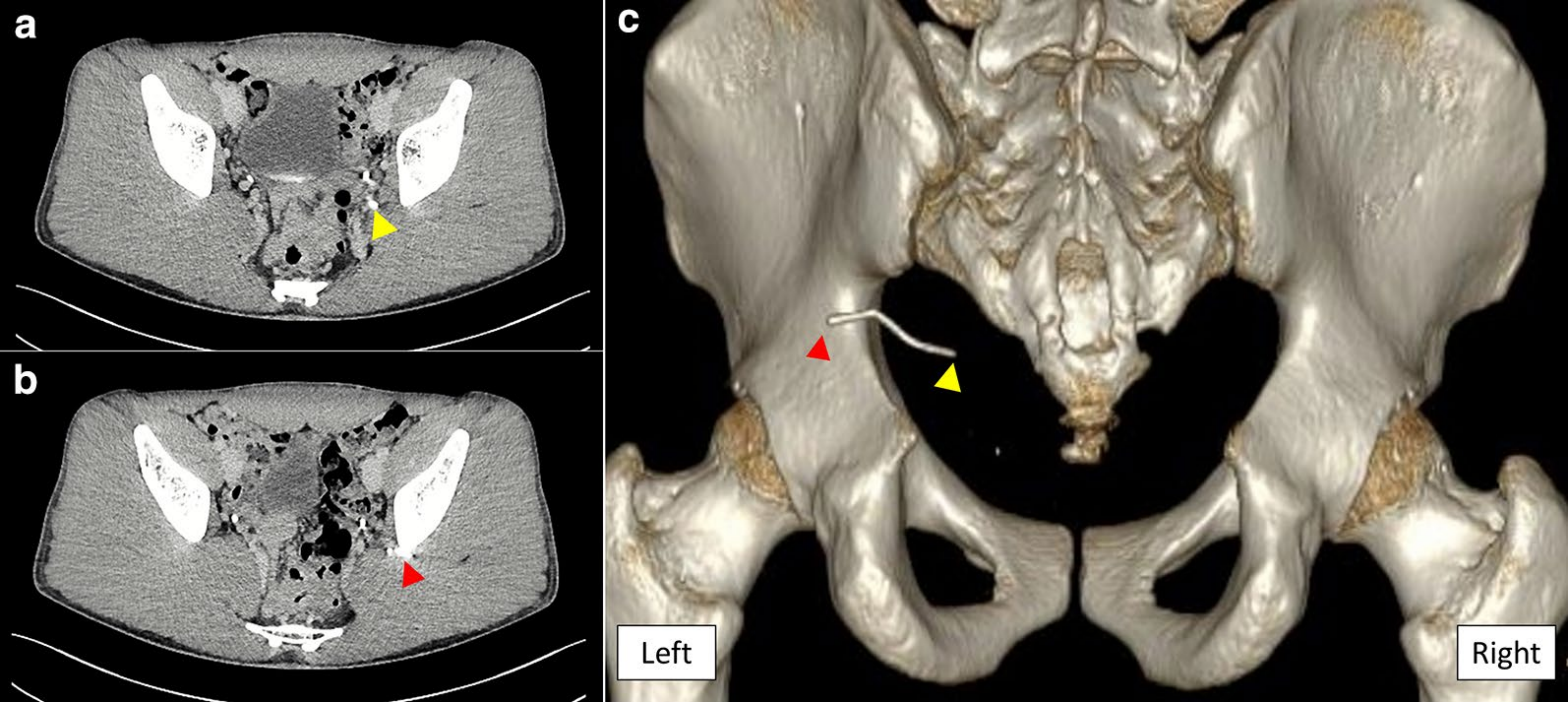
Computed tomography of the abdomen. **a** The tip of the needle (blue arrowhead) seems to reach the pelvic cavity through the retroperitoneum. **b** The stump of the needle (red arrowhead) is located in the gluteus. **c** Three-dimensional reconstructed image seen from the back clearly shows a linear, hyperdense foreign body in the gluteus

On examination, the patient’s height was 174 cm, body weight was 68 kg, and body mass index was 22.5 kg/m^2^. The abdomen was soft and flat with no tenderness. The insertion point of the needle on the left hip could not be identified. It was difficult to touch or feel the aberrant needle. The laboratory data on admission revealed no abnormal findings. Abdominal X-ray examination showed a thin, 40-mm-long, metallic foreign body resembling a needle used for acupuncture treatment (Fig. 1). Computed tomography (CT) of the abdomen showed a linear, hyperdense, foreign body in the gluteus. However, it was unclear whether the tip of the needle reached the pelvic cavity through the retroperitoneum. There was no evidence of free air, abscess formation, or migration of the foreign body into the intestine or vessels (Fig. 2a–c and Supplementary file).

It was reported that needle broken was very rare acupuncture adverse effect in which frequency was 0.001%, but also reported that all of them had need to treatment [2]. Although prompt foreign body removal was needed, in addition to the CT findings described above, the physical findings not reminiscent of acute abdomen suggested that there was no need to perform emergency surgery. It was considered that the best approach was to remove the foreign body safely and minimally invasively. The removal of the foreign body via an approach from the body surface by an orthopedic surgeon was initially discussed. However, it was expected to be difficult to identify the foreign body via the body surface approach because the stump of the needle was located in the middle of the gluteus. Smooth devices such as wire, pins, and needles have the potential to migrate to distant anatomical sites [4, 5]. Moreover, an incision in the gluteus muscle may have reduced the patient’s athletic ability. Consequently, it was considered more appropriate to use a transabdominal approach to remove the foreign body that was agreed by the patient, although there is some risk of repairing abdominal organs. The CT findings indicated that the foreign body would be visually recognized by an approach to the retroperitoneum similar to that used for lateral lymph node dissection in rectal cancer surgery. As a corroded needle might be fragile and fragment during removal [10], and retained needle fragments may cause abscess formation [3], it was finally decided that the aberrant needle would be laparoscopically removed under X-ray fluoroscopy guidance. Although it was believed that the foreign body could be found by laparoscopic approach, but in case it was difficult to laparoscopically confirm, we prepared for the conversion to laparotomy to direct search and palpate it from the abdominal cavity, which may help identify the foreign body. There are previous reports of laparoscopic removal of a pelvic foreign body [5, 11]. Liu et al. [12] reported the laparoscopic removal of a broken acupuncture needle from the retroperitoneum. Although this previous case differs from the present case in that it was not an intrapelvic foreign body and it was removed by the transretroperitoneum approach rather than the transabdominal approach, it still reports the merits of laparoscopic removal of an aberrant acupuncture needle. The clinical significance of our transabdominal approach utilizing laparoscopy can be the most reasonable and minimally invasive strategy in visual observation of abdominal organs at removal procedure.

**Fig. 3:**
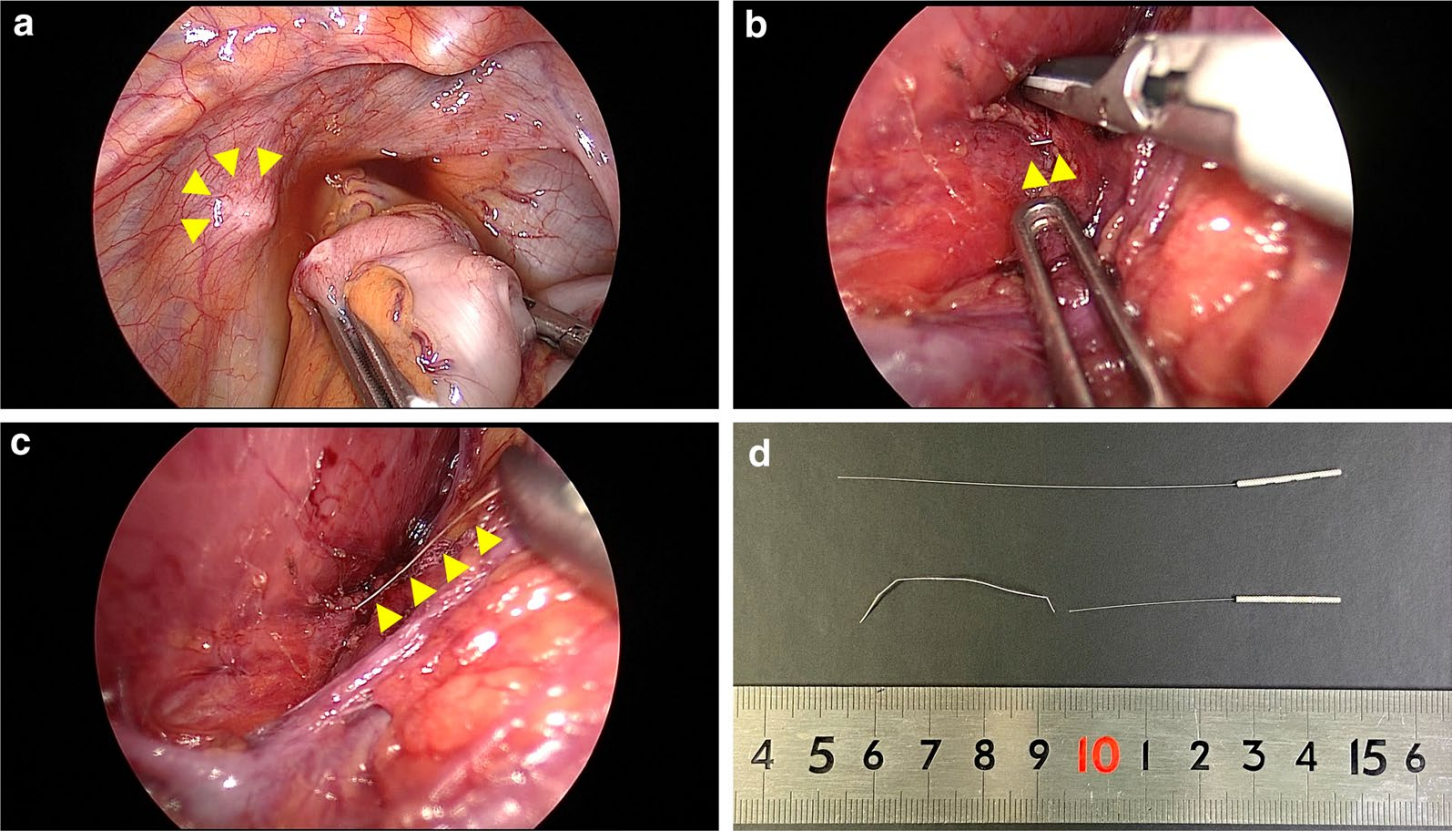
Operative findings and the extracted needle. **a** The peritoneum surrounding the needle has granulomatous changes due to inflammation. **b** The needle is identified where it enters the levator ani muscle near the arch of the tendon and enters the obturator internus muscle. **c** The needle is easily removed by grasping it directly with a needle holder. **d** The length of the aberrant needle is 40 mm, which is consistent with the preoperative imaging. The upper image shows an example of the same type of acupuncture needle. The lower image shows the retained aberrant needle that was extracted in laparoscopic surgery and the broken root part of the needle that was provided by the acupuncturist

The patient was placed in supine position. Five trocars were placed: one above the navel for the laparoscopy (12 mm), one in each of the upper and lower left abdominal quadrants (5 mm), one in the upper right abdominal quadrant (5 mm), and one in the lower right abdominal quadrant (12 mm). Although X-ray fluoroscopy confirmed that the aberrant needle was located in the gluteus, the needle could not be felt with the laparoscopic forceps, as the peritoneum surrounding the needle had granulomatous changes due to inflammation (Fig. 3a). Therefore, the retroperitoneum was further dissected to search for the needle. While identifying the anatomical structures with the approach used in lateral lymph node dissection, the needle was identified entering the levator ani muscle near the arch of the tendon and entering the obturator internus muscle (Fig. 3b). Because of its flexibility, the needle was easily removed by grasping it directly with a needle holder (Fig. 3c). All blood vessels and nerves were preserved. The length of the aberrant needle was 40 mm, which was consistent with the preoperative imaging (Fig. 3d). X-ray fluoroscopy confirmed that there was no residual foreign body. Eight days have passed from the acupuncture treatment to the removing of the needle on the surgery.

The patient recovered without complications and was discharged on the 2nd postoperative day and quickly returned to competitive sport.

## Conclusions

Intrapelvic aberrant needles are rare and may cause life-threatening damage to multiple organs; therefore, prompt removal is warranted. When the foreign body remains in the gluteus with its tip touching intrapelvic organs, such as the rectum, it is critical to determine the best approach for its safe removal. Given the anatomical location of the foreign body and the patient’s background, laparoscopic removal was the best approach in the present case.

## Supplementary information

**Additional file 1:** Video version of the CT findings. 

## Data Availability

Data sharing is not applicable to this article, as no datasets were generated or analyzed.

## References

[1] Eisenberg DM, Davis RB, Ettner SL, Appel S, Wilkey S, Van Rompay M (1998). Trends in alternative medicine use in the United States, 1990–1997: results of a follow-up national survey. JAMA.

[2] Witt CM, Pach D, Brinkhaus B, Wruck K, Tag B, Mank S (2009). Safety of acupuncture: results of a prospective observational study with 229,230 patients and introduction of a medical information and consent form. Forsch Komplementmed.

[3] Gonzalez-Ojeda A, Rodriguez-Alcantar DA, Arenas-Marquez H, Sanchez Perez-Verdia E, Chavez-Perez R, Alvarez-Quintero R (1999). Retained foreign bodies following intra-abdominal surgery. Hepatogastroenterology.

[4] Fong YC, Lin WC, Hsu HC (2005). Intrapelvic migration of a Kirschner wire. J Chin Med Assoc.

[5] Kottmeier S, Born CT, Saul H (1993). Laparoscopic retrieval of a migrating intrapelvic pin: case report and review of the literature. J Trauma.

[6] Mima K, Sugihara H, Kato R, Matsumoto C, Nomoto D, Shigaki H (2018). Laparoscopic removal of an ingested fish bone that penetrated the stomach and was embedded in the pancreas: a case report. Surg Case Rep.

[7] Demir SC, Cetin MT, Ucünsak IF, Atay Y, Toksöz L, Kadayifçi O (2002). Removal of intra-abdominal intrauterine device by laparoscopy. Eur J Contracep Reprod Health Care.

[8] Watanabe T, Tomibayashi A (2012). Laparoscopic removal of an intraperitoneal foreign body introduced through the urethra: a case report (in Japanese with English abstract). Nihon Naisikyou Geka Gakkaizasshi (J Jpn Soc Endosc Surg).

[9] Chiu WK, Hsiao CW, Kang JC, Feng JJ, Chao PC, Jao SW (2007). Intrapelvic migration with long-term retention of a rectal thermometer: a case report. Clin Pediatr.

[10] Oya S, Yuasa N, Takeuchi E, Goto Y, Miyake H, Miyata K (2017). Excision of a metallic foreign body at the same time as a laparoscopic cholecystectomy -a case report- (in Japanese with English abstract). Nihon Rinsho Geka Gakkaizasshi (J Jpn Surg Assoc).

[11] Karaman I, Kafadar IH, Oner M, Halici M (2013). Intrapelvic pin migration after Salter innominate osteotomy and laparoscopic removal: a case report. J Pediatr Orthop B.

[12] Liu ZH, Wang HD, Xu X, Man LB (2019). Removal of a broken acupuncture needle in retroperitoneum by laparoscopy: a case report. BMC Surg.

